# Assessing capacity and readiness to manage NCDs in primary care setting: Gaps and opportunities based on adapted WHO PEN tool in Zambia

**DOI:** 10.1371/journal.pone.0200994

**Published:** 2018-08-23

**Authors:** Wilbroad Mutale, Samuel Bosomprah, Perfect Shankalala, Oliver Mweemba, Roma Chilengi, Sharon Kapambwe, Charles Chishimba, Mulenga Mukanu, Daniel Chibutu, Douglas Heimburger

**Affiliations:** 1 University of Zambia, School of Public Health, Lusaka, Zambia; 2 Centre for Infectious Disease Research in Zambia, Lusaka, Zambia; 3 Department of Biostatistics, School of Public Health, University of Ghana, Legon, Accra, Ghana; 4 Ministry of Health, Lusaka, Zambia; 5 Vanderbilt University, Nashville, Tennessee, United States of America; University of Miami, UNITED STATES

## Abstract

**Introduction:**

Sub-Saharan Africa is experiencing an epidemiological transition as the burden of NCDs overtake communicable diseases. However, it is unknown what capacity and gaps exist at primary care level to address the growing burden of NCDs. This study aimed to assess the Zambian health system’s capacity to address in NCDs, using an adapted WHO Essential Non Communicable Disease Interventions (WHO PEN) tool.

**Methodology:**

This was a cross-sectional facility survey in the three districts conducted from September 2017 to October 2017. We defined facility readiness along five domains: basic equipment, essential services, diagnostic capacity, counseling services, and essential medicines. For each domain, we calculated an index as the mean score of items expressed as percentage. These indices were compared to an agreed cutoff at 70%, meaning that a facility index or district index below 70% off was considered as ‘not ready’ to manage NCDs at that level. All analysis were performed using Stata 15 MP.

**Results:**

There appeared to be wide heterogeneity between facilities in respect of readiness to manage NCDs. Only 6 (including the three 1^st^ level hospitals) out of the 46 facilities were deemed ready to manage NCDs. Only the first level hospitals scored a mean index higher than the 70% cut off; With regard to medications needed to manage NCDs, urban and rural health facilities were comparably equipped. However, there was evidence that calcium channel blockers (p = 0.013) and insulin (p = 0.022) were more likely to be available in urban and semi-urban health facilities compared to rural facilities.

**Conclusion:**

Our study revealed gaps in primary health care capacity to manage NCDs in Zambia, with almost all health facilities failing to reach the minimum threshold. These results could be generalized to other similar districts in Zambia and the sub-region, where health systems remain focused on infectious rather than non-communicable Disease. These results should attract policy attention and potentially form the basis to review current approach to NCD care at the primary care level in Zambia and Sub-Saharan Africa.

## Introduction

It has been reported that Sub-Saharan Africa is experiencing an epidemiological transition which is related to the change from infectious to Non-infectious Diseases [1]. This has resulted from the change in life styles including rapid urbanization and westernization of lifestyles^1^. Fuelling this new epidemic is the observed decrease in physical activity, change in diet and better life expectance at birth [1,2]. It has been recognized that NCDs have overtaken communicable diseases (CDs) as the principal causes of mortality (54% compared to 36%) in many low-income countries [2,3].

HIV services have an impact on NCDs. As most people with HIV are living longer due to Antiretroviral treatment, they are exposed to the risk of NCDs with some related to metabolic side effects of ART medications [4]. In 2010, NCDs were formally recognized at the UN General Assembly as an important missing element in the Millennium Development Goals (MDGs) [4] and have now been recognized as a serious threat in the current Sustainable Development Goals (SDG) [5,6].

A recent study conducted jointly by the World Economic Forum and Harvard University showed that NCDs are likely to cost the world economy $47 trillion over the next 20 years, representing 75% of global gross domestic product (GDP) and surpassing the cost of the global financial crisis [3]. This is far higher when compared to an estimated cost of $11.4 billion a year, required for Low- and Middle Income Countries (LMICs) to implement effective strategies to prevent and treat NCDs [3].

In Zambia, NCDs are among the top 10 causes of mortality, and the current health strategic plan 2017–2021 has placed NCDs as a priority area for intervention, with the government and cooperating partners working to address this growing threat [6,7]. Though population level data on NCDs is not available in Zambia, routine data collected from hospitals have shown a 22% increase in the total number of NCDs cases between 2010 and 2012 in all age groups [8]. In the same period, cases of hypertension seen in the outpatient department (OPD) increased by 39% for all age groups. Cancer cases seen at the country’s only Cancer Diseases Hospital (CDH) increased from 1282 in 2010 to 3021 in 2014, exhibiting an increase of over 50%.

## Limitations of NCD research conducted in Zambia

There are very few studies, which have addressed NCDs in Zambia. Most of the studies were conducted in urban settings and predominantly descriptive in nature. The main focus has been disease specific conditions such as diabetes, hypertension and cervical cancer. Only one study addressed the health system responsiveness to NCDs [9].

To comprehensively address NCDs, a systems approach is required [10]. The health system approach helps to understand how the various elements of a system can hinder or facilitate service delivery in general and specifically how these building blocks can impact services related to NCDs at the primary health care level. Such an approach would help to identify the available resources and gaps which can inform interventions which target the whole system, leveraging resources while addressing crucial gaps in the overall health system.

In this study, we applied a health systems approach to assess the Zambian health system’s capacity to address NCDs, using an adapted WHO Essential Non-Communicable Disease tool (WHO PEN) at primary care level.

## Methodology

### Study settings

The study was conducted in three districts namely, Chongwe, Kafue and Luangwa. These districts make up all rural and peri-urban districts of Lusaka Province. These districts comprise approximately 48 primary health facilities, which were formerly intervention sites for Better Health Outcomes through Mentorship and Assessment (BHOMA) health system strengthening intervention [9]. The BHOMA project which closed in December 2015 was a randomized community intervention focusing on strengthening the health system capacity to address infectious diseases such as HIV and tuberculosis, with very little emphasis on NCDs [11]. All eligible health facilities were included in the study.

### Study design

This was a cross-sectional facility survey in the three districts conducted from September 2017 to October 2017. The target was primary health care facilities. However, we also included three ‘first level facilities’ in the districts, as they also provide some primary care services, as a benchmark.

### Data collection

We adapted the WHO PEN tool through consultative meetings with key stakeholders in the Zambian health system including management at the Ministry of Health, district health directors, health facility managers and frontline workers. The stakeholders were availed the tool for review before the scheduled date of the interview. After the consultative meetings, a three-day workshop was held with all the stakeholders that had been interviewed to reach a consensus on the modification that would be made to the tool. Following the deliberations from the workshop, the modified tool was then circulated to the stakeholders for final approval. The tool was then piloted at three facilities not earmarked for assessment, and the final accepted tool used for data collection for this study.

We then used the adapted PEN tool to collect data from eligible health facilities. Two research assistants with health profession background, were trained in data collection for 2 days. Data collection was conducted between August-October 2017 and covered all eligible health facilities.

### Statistical analysis

We defined facility readiness along five domains: basic equipment, essential services, diagnostic capacity, counseling services, and essential medicines. For each domain, we calculated an index as the mean score of items expressed as percentage. For example, there were 13 equipment items on the survey, and if a facility had 5 functioning equipment items, the basic equipment index for that facility was calculated as 5*100/13 = 38.5%. The facility readiness index was then calculated as the average of domain indices. We display the indices by districts and by facilities. For the district level disaggregation, we considered the three 1^st^ level hospitals as a separate category. These indices were compared to an agreed cutoff at 70%, meaning that a facility index or district index below 70% off was considered as ‘not ready’ to manage NCDs at that level. We used Fisher’s exact test to assess differences in essential medicine availability by rural-urban status of health facilities. All analysis were performed using Stata 15 MP (StataCorp, College Station, Texas, USA).

### Ethical consideration

The study obtained ethics approval from the University of Zambia Biomedical Research Ethics Committee. All participants were provided with study information before signing the consent form. Confidentiality was assured during data collection and publications.

## Results

### Health facility characteristics

A total of 46 primary health care facilities were assessed. Of these, 21 were in Chongwe, 15 in Kafue, 10 in Luangwa districts (Table 1). There were three 1st level hospitals of which two were in Luangwa district and one in Chongwe district (Table 1). When assessed by rural vs. urban setting as classified by the national census system, 38 (83%) of the health facilities were located in rural settings (Table 1).

**Table 1:** Characteristics of health facilities.

Characteristics	Number of health facilities	% of total
**Ownership**		
Public	45	97.8
Faith-based	1	2.2
**Type**		
Health Centre	36	78.3
Zonal Health Centre	7	15.2
Mission/Level 1 Hospital	3	6.5
**Setting**		
Rural	38	82.6
Urban	4	8.7
Semi-Urban	4	8.7
**District**		
Chongwe	21	45.7
Kafue	15	32.6
Luangwa	10	21.7
**Bed to stabilise patients**		
No	6	13
Yes	40	87
**Total**	46	100

### Health facility readiness

**Fig 1:**
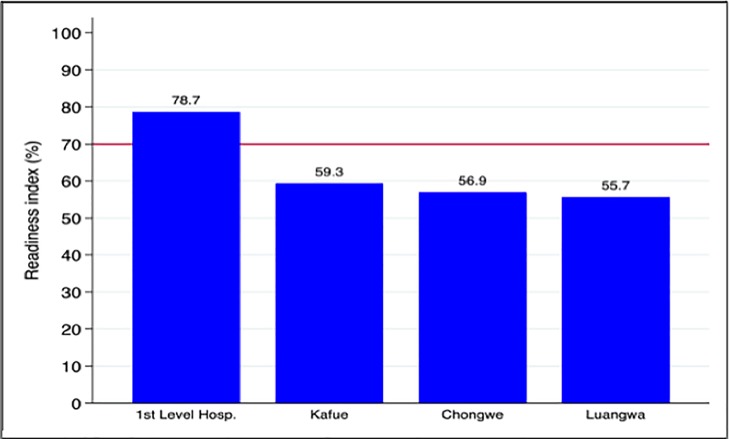
Readiness index by district. The horizontal red line indicates the cut off below which a district was considered as ‘not ready’ to manage NCDs.

**Fig 2:**
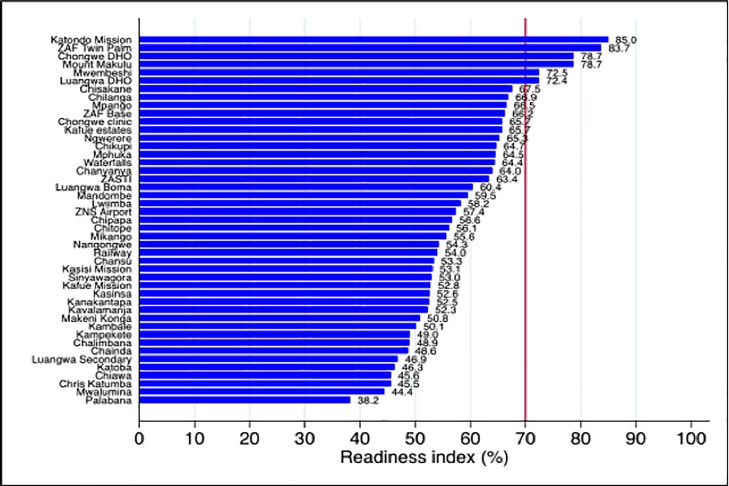
Readiness index by facilities. The vertical red line indicates the cut off below which a facility was considered as ‘not ready’ to manage NCDs.

**Fig 3:**
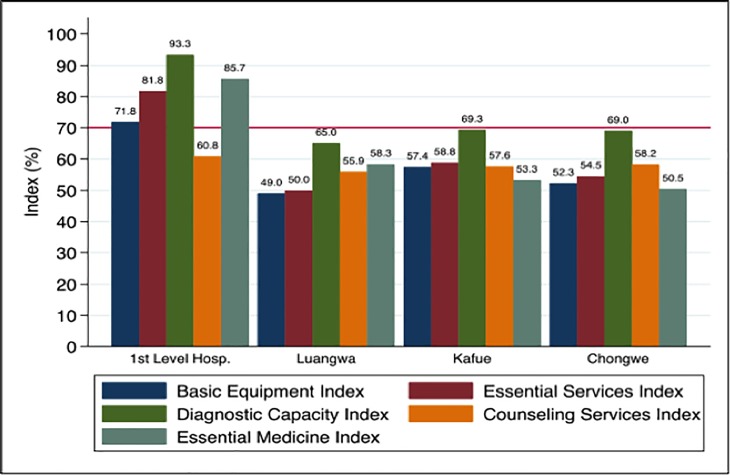
Domain-specific index by district. The red horizontal line indicates the cut off below which a district was considered as ‘not ready’ for that domain to manage NCDs.

The 1^st^ level hospitals (Katondwe, Chongwe, Luangwa DHO) had the highest readiness index (78.7) followed by Kafue (59.3), Chongwe (56.9), and Luangwa (55.7) (Fig 1). There appeared to be wide heterogeneity between facilities in respect of its readiness to manage NCDs (Fig 2). Only 6 (including the three 1^st^ level hospitals) out of the 46 facilities were deemed ready to manage NCDs (Fig 2). Except for diagnostic capacity, none of the districts were ready in terms of the other four domains to manage NCDs (Fig 3).

With regard to medications needed to manage NCDs, urban and rural health facilities were comparably equipped except calcium channel blockers (p = 0.013) and insulin (p = 0.022) were more likely to be available in urban and semi-urban health facilities compared to rural facilities (Table 2).

**Table 2:** Difference in essential medicine availability by rural-urban status.

Essential medicine availability	Rural (N = 38)	Urban & Semi-Urban (N = 8)	Fisher's exact p-value
	n (%)	n (%)	
Beta Blockers	11 (29)	2 (25)	1.000
Antibiotics	36 (95)	8 (100)	1.000
Diuretics	21 (55)	6 (75)	0.440
ACE Inhibitors	1 (3)	2 (25)	0.074
Steroids	37 (97)	7 (88)	0.321
Oral antihyperglycemics	17 (45)	4 (50)	1.000
Analgesics	30 (79)	6 (75)	1.000
Salbutamol inhaler	28 (74)	6 (75)	1.000
Epileptic drugs	15 (40)	3 (38)	1.000
Calcium channel blockers	6 (16)	5 (63)	**0.013**
Adrenaline (injection)	26 (68)	7 (88)	0.409
Aspirin	25 (66)	6 (75)	1.000
Insulin (long acting)	4 (11)	4 (50)	**0.022**
Insulin (soluble)	7 (18)	3 (38)	0.344
Statins (lovastatin or simvastatin)	0	0	
Sodium chloride infusion	33 (87)	6 (75)	0.587
Glyceryl trinitrate	2 (5)	1 (13)	0.444
Clotrimoxazole	35 (92)	7 (88)	0.548
Promethazine injection	34 (89)	8 (100)	1.000
Glucose injectable	31 (82)	7 (88)	1.000
Aminophylline	29 (76)	6 (75)	1.000

As summarized in Fig 1, only the first level hospitals scored a mean index higher than the 70% cut off; the rest of the district facilities’ scores were 59.3, 56.9 and 55.7 for Kafue, Chongwe and Luangwa, respectively.

Readiness index scores disaggregated by individual health facilities are summarized in Fig 2. The least ready facility scored 38.2, while three primary care facilities passed the 70% threshold.

## Discussion

Our study is the first in Zambia to apply a health systems approach and the WHO PEN tool to evaluate the readiness of the Zambia health system to manage NCDs. The study has revealed that nearly all primary health facilities studied could not meet the minimum threshold to manage NCDs in line with WHO recommendations [12].

First-level hospitals generally performed better than lower-level primary health facilities. We disaggregated the hospitals because the tool is designed to assess primary level care, while the hospitals are essentially at a higher secondary level. Nonetheless, the assessed hospital facilities barely made it despite being higher level service providers; in fact, their counseling services’ mean index was below par at 60.8.

There were no significant differences across the districts in terms of capacity and readiness, although Kafue generally had higher scores compared to Chongwe or Luangwa. One reason could be that it is relatively more urban than the other districts.

We also noted poor performance across the domains assessed in the three districts, including services that did not require equipment such as patient and family counseling. Yet the same facilities are providing counseling services to HIV patients and families. This shows how the current health system has been shifted toward HIV Care, completely ignoring other disease that a prevalent such as NCDs [9].

We used the WHO PEN tool, which we adapted to the Zambian context to rank the performance of the facilities. Our results showed that this tool was adequate to capture the different domains of health system and was able to distinguish performance across the domains and districts. This tool is therefore useful and could be applied across the Zambia health sector at first- and second-level hospitals. In addition, this tool could be applicable to other LMICs to map their efforts to address NCD goals and defining priorities [13,14]. One challenge with the tool however, was the variation brought about by the presence or absence of specific variables which were weighted similarly without regard to their relative importance. Future assessments could be more objective if the tool variables are weighted against relative importance in that domain; for example, equipment needed for primary level care such as blood pressure machines could have a greater weight than those needed to provide secondary or tertiary care services.

Our findings are not unique to Zambia. Similar studies conducted in other LMICs have shown similar deficiencies in health system infrastructure, workforce capacity, surveillance, planning, policy, and program management [15,16]. The major difference with our study was the use of a locally adapted tool and focusing on local health systems rather than a global overview, which often ignore country specific context [17,18].

Our study has prepared the way for strategic interventions designed to address identified gaps. The next logical step is for us to work on specific pragmatic interventions to address the identified gaps. A study conducted in Vietnam using the WHO PEN approach, and addressing similar gaps resulted in marked improvements in trained health service providers; availability of essential equipment, supplies and medicines; functional referral systems; and use of monitoring tools [16]. We recommend that similar studies be conducted in Zambia, especially in urban settings were the context might be different from rural sites.

Our study has several limitations. The major limitation of our study is that it focused on the supply side of the health system and gave less consideration for the demand-side gaps, which could be important when designing interventions to address the gaps identified [19].

The study was cross-sectional and hence we cannot attribute the gaps to specific causes and we are not able to explain linkages across the building blocks, which is often anticipated in complex health systems [20,21,22].

The study relied on verbal responses in some domains where it was not possible to verify the information. This can bias our results with the potential that our findings could underestimate the gaps due to the potential for responders to give positive outlook for their facilities. It is also possible that the bias could be reversed if respondents wanted to exaggerate their facilities’ gaps in order to draw attention.

## Conclusion

Our study revealed gaps in primary health care capacity to manage NCDs in Zambia, with almost all health facilities failing to reach the minimum threshold. These results could be generalized to other similar districts in Zambia and the sub-region, where health systems remain focused on infectious rather than non-communicable Disease. These results should attract policy attention and potentially form the basis to review current approach to NCD care at the primary care level in Zambia and Sub-Saharan Africa.
